# Comprehensive miRNA expression profiling in human T-cell acute lymphoblastic leukemia by small RNA-sequencing

**DOI:** 10.1038/s41598-017-08148-x

**Published:** 2017-08-11

**Authors:** Annelynn Wallaert, Wouter Van Loocke, Lucie Hernandez, Tom Taghon, Frank Speleman, Pieter Van Vlierberghe

**Affiliations:** 1 Center for Medical Genetics Ghent, Ghent University, Ghent, Belgium; 2 Cancer Research Institute Ghent, Ghent, Belgium; 3 University Paris Diderot and Hospital Saint-Louis, U944 INSERM Paris, France; 4 Department of Clinical Chemistry, Microbiology and Immunology, Ghent University, Ghent, Belgium

## Abstract

T-cell acute lymphoblastic leukemia (T-ALL) is a genetically heterogeneous disease that can be classified into different molecular genetic subtypes according to their mRNA gene expression profile. In this study, we applied RNA sequencing to investigate the full spectrum of miRNA expression in primary T-ALL patient samples, T-ALL leukemia cell lines and healthy donor thymocytes. Notably, this analysis revealed that genetic subtypes of human T-ALL also display unique miRNA expression signatures, which are largely conserved in human T-ALL cell lines with corresponding genetic background. Furthermore, small RNA-sequencing also unraveled the variety of isoforms that are expressed for each miRNA in T-ALL and showed that a significant number of miRNAs are actually represented by an alternative isomiR. Finally, comparison of CD34^+^ and CD4^+^CD8^+^ healthy donor thymocytes and T-ALL miRNA profiles allowed identifying several novel miRNAs with putative oncogenic or tumor suppressor functions in T-ALL. Altogether, this study provides a comprehensive overview of miRNA expression in normal and malignant T-cells and sets the stage for functional evaluation of novel miRNAs in T-ALL disease biology.

## Introduction

T-cell acute lymphoblastic leukemia (T-ALL) is an aggressive hematological malignancy that is classified into different genetic subtypes based upon the aberrant expression of specific transcription factor oncogenes (*TAL*, *TLX1*, *TLX3* or *HOXA*) or the arrest at a specific stage of T-cell differentiation (immature T-ALL)^1–4^. Notably, these molecular subgroups are characterized by unique mRNA and long non-coding RNA expression signatures, which partially reflect their putative cell of origin^1, 2, 5^.

MicroRNAs (miRNAs) are short non-coding RNAs that function as post-transcriptional repressors of specific target genes^6, 7^. Several studies have previously described a role for miRNAs in malignant T-cell transformation, including the identification of both an oncogenic (*miR-19b*, *mir-20a*, *miR-26a*, *miR-92* and *miR-223*)^8^ as well as a tumor suppressor (miR-150, *miR-155*, *miR-200* and *miR-193b-3p*)^9, 10^ miRNA network involved in T-ALL disease biology. However, studies that addressed the role of miRNAs in human T-ALL have largely been focused on previously recognized miRNA molecules as they consistently used RT-qPCR or microarrays as detection platforms.

More recently, small RNA-sequencing emerged as a more comprehensive technology that enables unbiased detection of the full spectrum of small RNA molecules. In addition, it also provides information on specific isoforms that differ from canonical miRNAs by the addition or deletion of one or more nucleotides at the 5′ or 3′ end of the miRNA^11, 12^. Notably, this heterogeneity in miRNA sequences, which is thought to result from RNA-editing, exonuclease activity or imprecise cleavage by DICER or DROSHA (ribonucleases involved in the miRNA processing)^13^, could be functionally relevant as shown for a number of miRNAs^14–17^.

Here, we used small RNA-sequencing to study the full spectrum of miRNAs that are expressed in human T-ALL samples. We demonstrate, for the first time, that molecular genetic subtypes of human T-ALL are characterized by unique miRNA expression signatures, delineate the pattern of miRNA isoforms that are expressed in malignant T-cells and use small RNA sequencing profiles of normal T-cell subsets to identify novel putative oncogenic or tumor suppressive miRNAs in the context of human T-ALL.

## Results

### Small RNA-sequencing of T-ALL patient samples, healthy donor thymocytes and T-ALL cell lines

**Figure 1:**
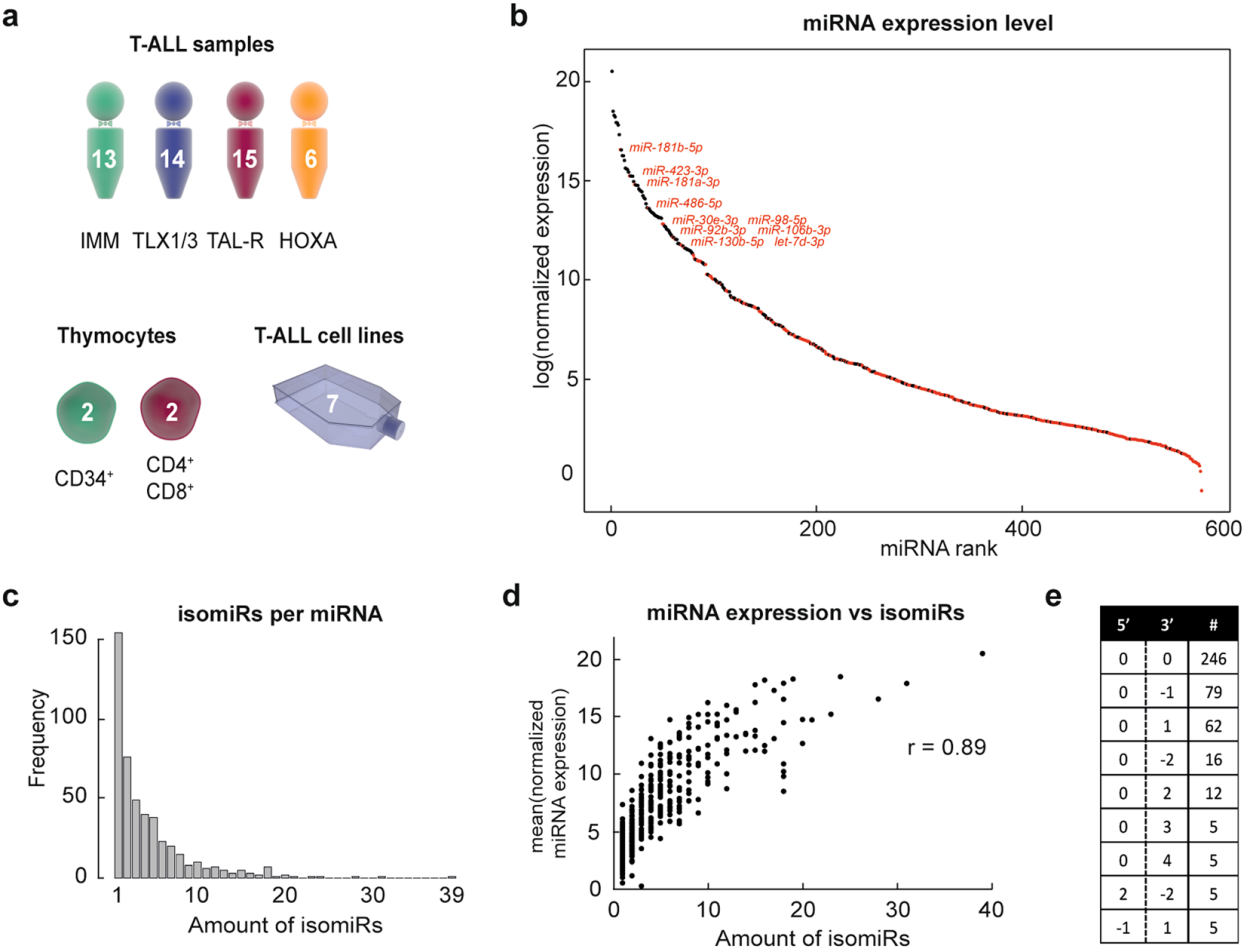
Small RNA-sequencing of T-ALL samples, healthy thymocytes and cell lines detects novel T-ALL miRNAs and isomiRs. (**a**) Overview of samples profiled by small RNA-sequencing. (**b**) Dot plot representing the mean normalized expression levels of all 574 miRNAs detected by small RNA-sequencing of the 48 T-ALL patient samples. Each dot represents one miRNA and the miRNAs are ranked from highest to lowest mean expression. Black dots are miRNAs that were already detected by a qRT-PCR platform from previous studies. Red dots are the novel miRNAs detected in T-ALL samples. (**c**) Bar plot visualizing the distribution of the miRNAs by means of the amount of isomiRs they are represented by. (**d**) Correlation plot between the mean expression level of the miRNAs and their amount of isomiR forms. (**e**) Table representing the isomiR form that was represented by the highest expression level for each of the detected miRNAs. The first column denotes the 5′ overhangs or deletions, the second column the 3′ overhang or deletions of the isomiR in comparison to the canonical miRNA. The third column shows the amount of miRNAs of which the highest expressed miRNA had that isomiR form. (Graphics from www.somersault1824.com).

To study the full spectrum of miRNAs involved in normal and malignant T-cell development, we performed small RNA-sequencing on 48 primary T-ALL patient samples of different T-ALL subgroups (13 immature, 14 *TLX1* ^+^ or *TLX3* ^+^, 15 *TAL*-rearranged and 6 *HOXA*-overexpressing T-ALL samples), CD34^+^ and CD4^+^CD8^+^ normal thymocyte subsets from healthy donors and a panel of 7 T-ALL cell lines (Fig. 1a). While 1816 miRNAs were initially detected in the total panel of T-ALL patient samples, we further only considered the 574 miRNAs for which four reads were present in at least 60% of patients from one T-ALL subtype (Fig. 1b).

Given that we previously performed miRNA expression profiling using the same T-ALL patient cohort using an RT-qPCR approach^10^, we were able to directly compare the results from the RT-qPCR study with our small RNA-sequencing effort. Using a similar background selection as mentioned above, 283 miRNAs were previously detected by RT-qPCR^10^, which converted into 248 unique miRNAs that are currently annotated in the most recent version of miRBase^18^. From these, 198 miRNAs were also detected using our current small RNA-sequencing approach. Therefore, the RT-qPCR approach still detected 50 miRNAs that were not identified in the small RNA-sequencing dataset. In contrast, small RNA sequencing detected 259 miRNAs, that were not included in a previous RT-qPCR analysis from the same T-ALL patient population^10^.

In Fig. 1b and Supplementary Fig. S1, the mean expression values for the 574 miRNAs, detected by small RNA-sequencing in primary T-ALL patients, are plotted, with the black dots representing the 280 miRNAs that were previously identified by RT-qPCR and the red dots representing the miRNAs detected for the first time in human T-ALL. Most of the formerly known miRNAs reside amongst the most highly expressed in human T-ALL (Fig. 1b). However, some of the newly identified miRNA transcripts in T-ALL also show a very high expression pattern (the top 10 of highest expressed novel miRNAs are annotated in Fig. 1b), suggesting that they might possess oncogenic potential in the context of T-ALL disease biology. Nevertheless, the average expression level of most novel T-ALL miRNAs that were exclusively detected by small RNA-sequencing is median to low.

Small RNA-sequencing also enables the detection of so-called isomiRs, i.e. miRNA isoforms that deviate from the canonical sequence by one or a few nucleotide(s)^11, 12^. In our small RNA-sequencing dataset, we identified 2139 isomiRs covering 481 different miRNAs, losing some very low expressed miRNAs from the analysis above. Although 154 miRNAs were only represented by one isomiR, some others showed expression of more than 10 different isomiR forms (Fig. 1c). We observed a positive correlation between the number of isomiRs detected for a specific miRNA and its expression level in human T-ALL (r = 0.89, Fig. 1d). For example, we detected 39 different isomiR forms for miR-181a-5p, the miRNA that shows the highest average expression in T-ALL. Remarkably, for 106 out of 481 miRNAs, the canonical miRNA was not expressed in our patient series. In addition, for only half of the miRNAs (246 out of 481), the canonical isoform showed the highest expression level, suggesting that a substantial amount of miRNAs are mainly represented by alternative isomiRs. The distribution of isomiRs that show the highest expression for each miRNA is shown in Fig. 1e.

### Small RNA-sequencing reveals a subtype specific expression pattern of miRNAs in human T-ALL

**Figure 2:**
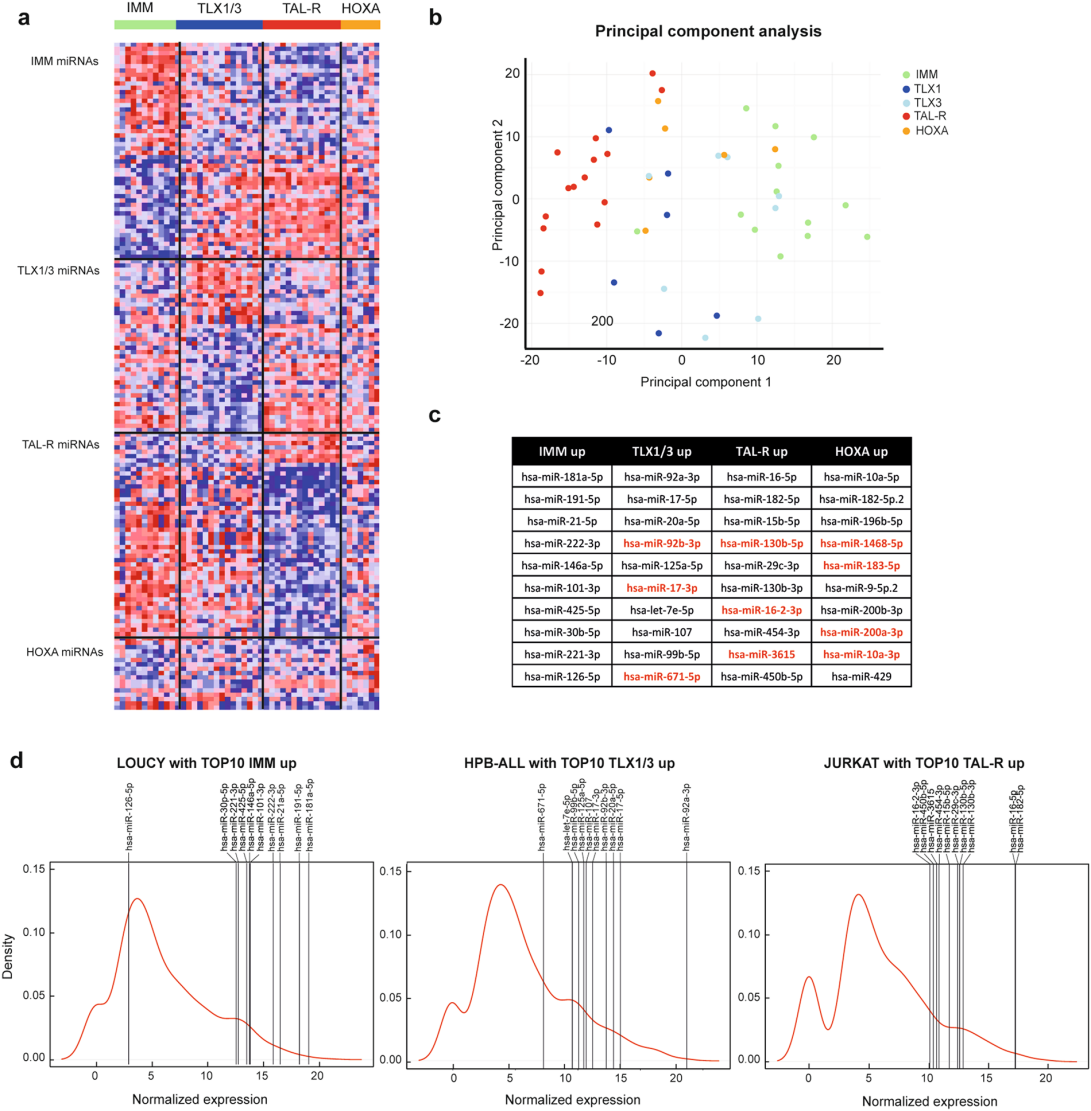
Small RNA-sequencing of primary T-ALL samples reveals a subtype specific expression pattern of miRNAs. (**a**) Heatmap representing the top 50 most significantly up- or downregulated miRNAs per subgroup in comparison to the other subgroups (adjusted p-value < 0.05). **(b**) PCA-plot showing the distribution of the patient samples. The different colors denote patient samples from a different subgroup. **(c**) Table representing the selection of 10 miRNAs per subgroup. These were the highest expressed miRNAs that were significantly upregulated in that subgroup compared to the other subgroups. MiRNAs denoted in red were not detected by a previously used qRT-PCR platform. (**d**) Density plots representing the distribution of the miRNA expression in the LOUCY, HPB-ALL and JURKAT cell line. Vertical bars show the expression level of the top 10 miRNAs selected for the subgroup these cell lines represent. LOUCY represents the immature T-ALL subgroup, HPB-ALL the *TLX1/3* subgroup and JURKAT the *TAL-R* subgroup.

Previous studies have convincingly shown that molecular genetic subtypes of human T-ALL display unique mRNA^1, 2^ and lncRNA^5^ expression signatures. Here, we used the 574 miRNAs detected by small RNA-sequencing to define subtype specific miRNA expression signatures in human T-ALL (adjusted p-value < 0.05; Fig. 2a; Supplementary Table S1). Principal Component Analysis confirmed that the most pronounced differences in miRNA expression are observed between immature and *TAL-R* T-ALL patient samples (Fig. 2b). Of note, although *TLX1* and *TLX* *3* patients have previously been considered as one genetic entity, miRNA based clustering did reveal some differences between members of the TLX subtype (Fig. 2b). For example, the miRNA expression signature of a specific subset of TLX3 positive T-ALLs is clearly more related to immature T-ALL, which was not observed for any of the TLX1 positive leukemias (Fig. 2b). The 10 miRNAs that show the highest expression level in each of the genetic subtypes are shown in Fig. 2c. The miRNAs, which were not previously detected by the RT-qPCR platform, are depicted in red (Fig. 2c).

As mentioned above, small RNA-sequencing was also performed on a panel of 7 human T-ALL cell lines. These *in vitro* model systems reflect most of the different genetic subtypes of human T-ALL and included *TLX1*/*3* positive (ALL-SIL, DND41 and HPB-ALL), *TAL-R* positive (PF-382, JURKAT and KE-37) and immature/*HOXA* overexpressing (LOUCY) tumor lines. Notably, based on the miRNAs represented in Fig. 2a, most T-ALL cell lines clustered separate from the T-ALL patient samples, with the exception of LOUCY, which clustered together with immature T-ALL patient samples, and ALL-SIL (Supplementary Fig. S2). Nevertheless, subtype specific miRNAs, which were identified in primary T-ALL patient samples, also showed high expression in the T-ALL cell lines from their corresponding genetic subtype (Fig. 2d and Supplementary Fig. S3). Therefore, the subtype specific tumor lines can be used as valuable *in vitro* tools to evaluate the role of specific miRNAs in the pathogenesis of this disease.

### MiRNA profiling of normal thymocyte subsets reveals oncogenic subtype specific miRNAs

**Figure 3:**
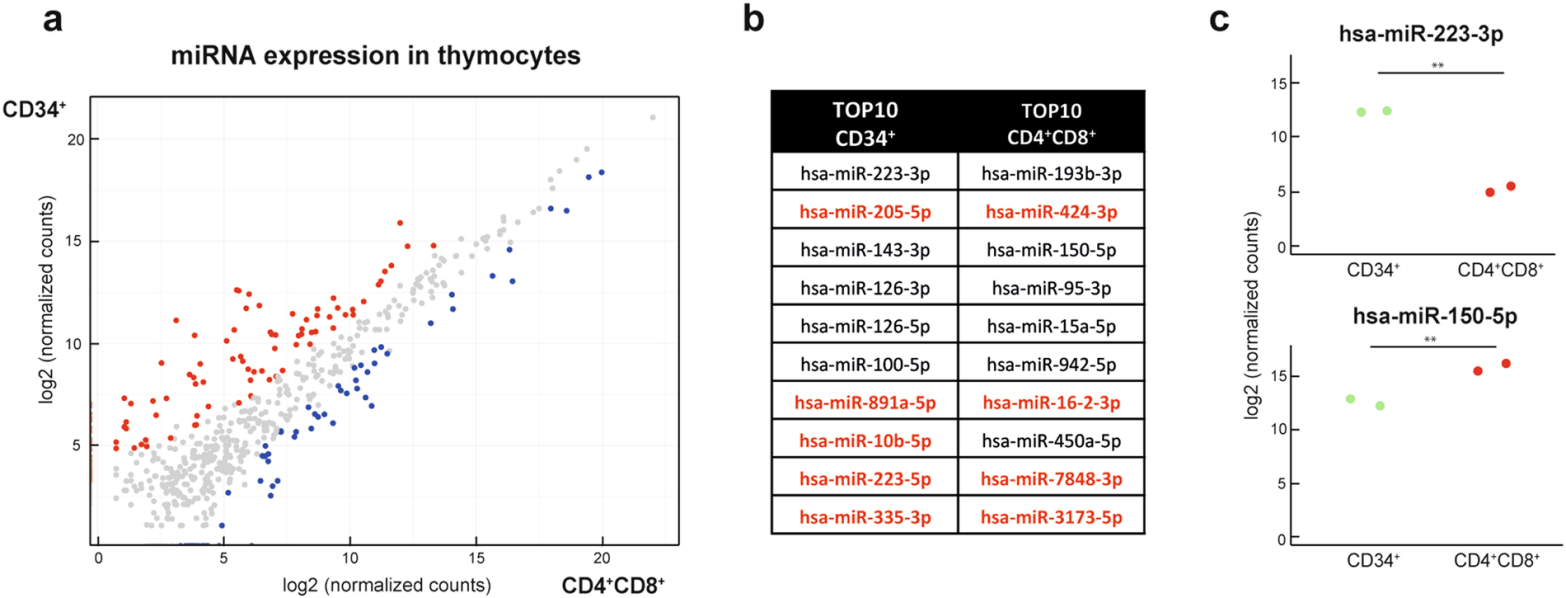
MiRNA profiling of healthy thymocyte subsets reveals different miRNA expression profiles between CD34^+^ and CD4^+^CD8^+^ subsets. (**a**) Diagonal plot showing the expression of the miRNAs in the different thymocyte subsets. Red dots represent miRNAs that are significantly higher expressed in the CD34^+^ subset, blue dots are the miRNAs significantly higher expressed in the CD4^+^CD8^+^ subset. (**b**) Top 10 most significantly upregulated miRNAs for the CD34^+^ subset and for the CD4^+^CD8^+^ subset. MiRNAs denoted in red were not detected by a previously used qRT-PCR platform. (**c**) Dot plots of two representative miRNAs for the subsets. **Significant difference with an adjusted p-value < 0.001.

Small RNA-sequencing was also performed on CD34^+^ and CD4^+^CD8^+^ normal thymocyte samples from two healthy donor controls. First, DESeq2 analysis revealed 190 miRNAs that show significant differential expression between these CD34^+^ and CD4^+^CD8^+^ normal T-cell subsets (126 high in CD34^+^ and 64 high in CD4^+^CD8^+^; adjusted p-value < 0.05; Fig. 3a and Supplementary Table S2). The top ten most significant miRNAs are listed in Fig. 3b, with the miRNAs depicted in red those that were not covered in previous RT-qPCR analyses. Of note, small RNA-sequencing data were highly concordant between both donors for each subset (Fig. 3c and Supplementary Fig. S4).

**Figure 4:**
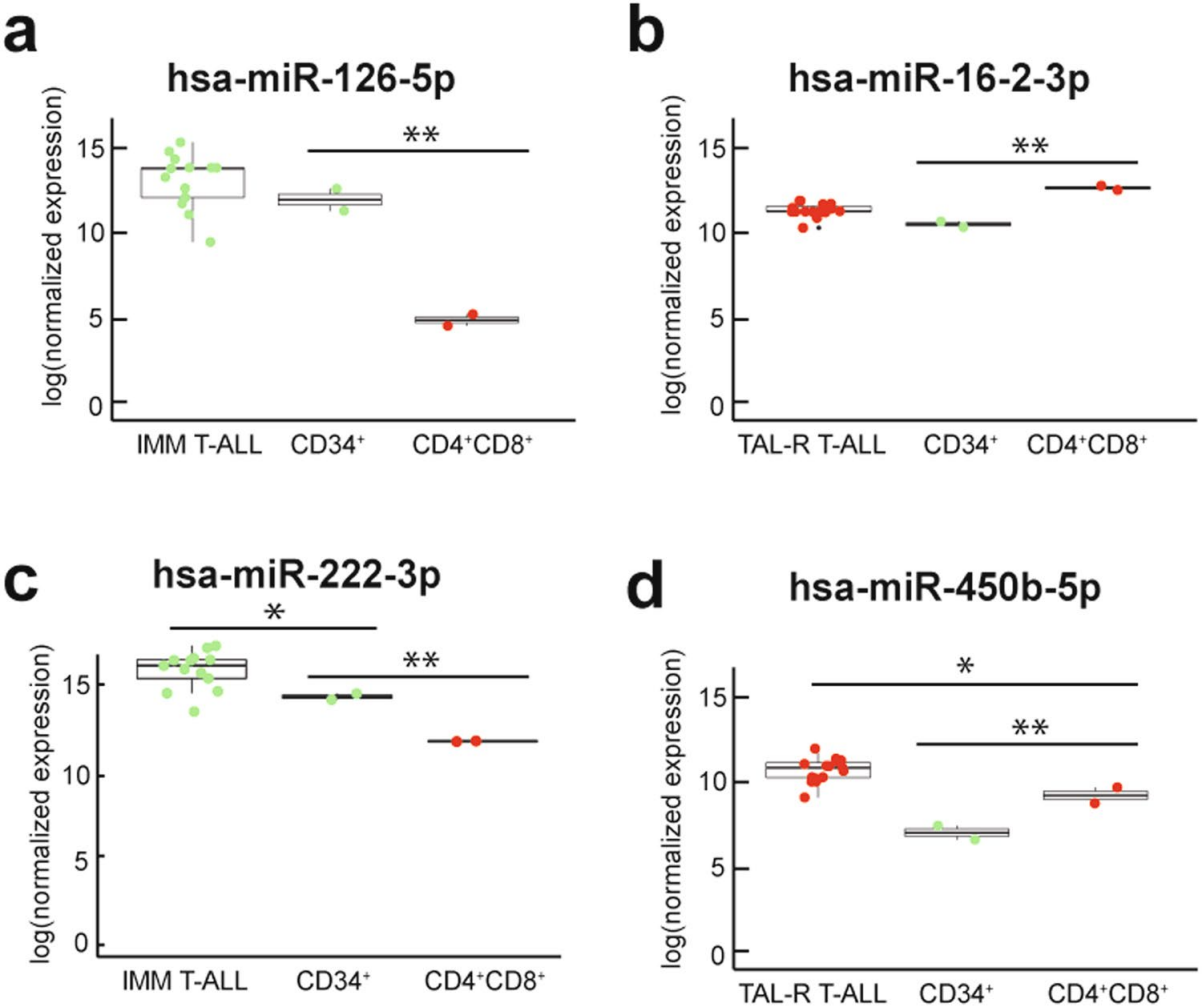
Subtype specific miRNAs can be either oncogenic or representative for the differentiation arrest of lymphoblasts. (**a** + **c**) Box plots showing the expression of the immature T-ALL specific miRNAs *hsa-miR-126-5p* and *hsa-miR-222-3p* in the immature T-ALL patients and the thymocyte subsets. (**b** + **d**) Box plots showing the expression of the *TAL-R* T-ALL specific miRNAs *hsa-miR-16-2-3p* and *hsa-miR-450b-5p* in the *TAL-R* T-ALL patients and the thymocyte subsets. *Significant difference with an adjusted p-value < 0.05; **significant difference with an adjusted p-value < 0.001.

Next, we integrated these miRNA expression data obtained from healthy donors with the subtype specific miRNAs that were identified in the primary T-ALL patient cohort. For example, 4 miRNAs (*hsa-miR-222-3p*, *hsa-miR-146a-5p*, *hsa-mir-221-3p* and *hsa-miR-126-5p*) from the top 10 immature T-ALL specific miRNAs (Fig. 2c) also showed significant higher expression in CD34^+^ vs. CD4^+^CD8^+^ T-cell subsets (Fig. 4a). Similarly, three miRNAs (*hsa-miR-16-5p*, *hsa-miR-16-2-3p* and *hsa-miR-450b-5p*) from the top 10 *TAL-R* specific miRNAs (Fig. 2c) are significantly upregulated in CD4^+^CD8^+^ T-cell subsets (Fig. 4b). Therefore, these miRNAs most probably reflect the specific T-cell maturation arrest associated with these molecular genetic subtypes of T-ALL and their respective cell of origin.

In order to identify miRNAs with putative oncogenic potential in specific T-ALL subgroups, we also performed differential expression analysis between immature T-ALLs and CD34^+^ thymocytes, and between *TAL-R* T-ALLs and CD4^+^CD8^+^ thymocytes, as these thymocyte subsets represent the stage of differentiation arrest during T cell development leading to these specific T-ALLs (Supplementary Tables S3 and S4). From the top ten immature specific miRNAs, three miRNAs (*hsa-miR-21-5p*, *hsa-miR-222-3p* and *hsa-miR-101-3p*) were significantly upregulated in the immature samples compared to the healthy control CD34^+^ samples. Remarkably, *hsa-miR-222-3p* was also significantly upregulated in the CD34^+^ subset, but its expression is further increased in immature T-ALLs (Fig. 4c). Similarly, three miRNAs (*hsa-miR-182-5p*, *hsa-miR-29c-3p* and *hsa-miR-450b-5p*), from the *TAL-R* subtype specific signature, show significant higher expression in the *TAL-R* T-ALLs as compared to their CD4^+^CD8^+^ normal counterparts. Here, *hsa-miR-450b-5p* was already higher expressed in the CD4^+^CD8^+^ double positive subset, but showed a further increase in activity in *TAL-R* rearranged leukemias (Fig. 4d).

### Small RNA-sequencing reveals novel putative oncogenic miRNAs in human T-ALL

**Figure 5:**
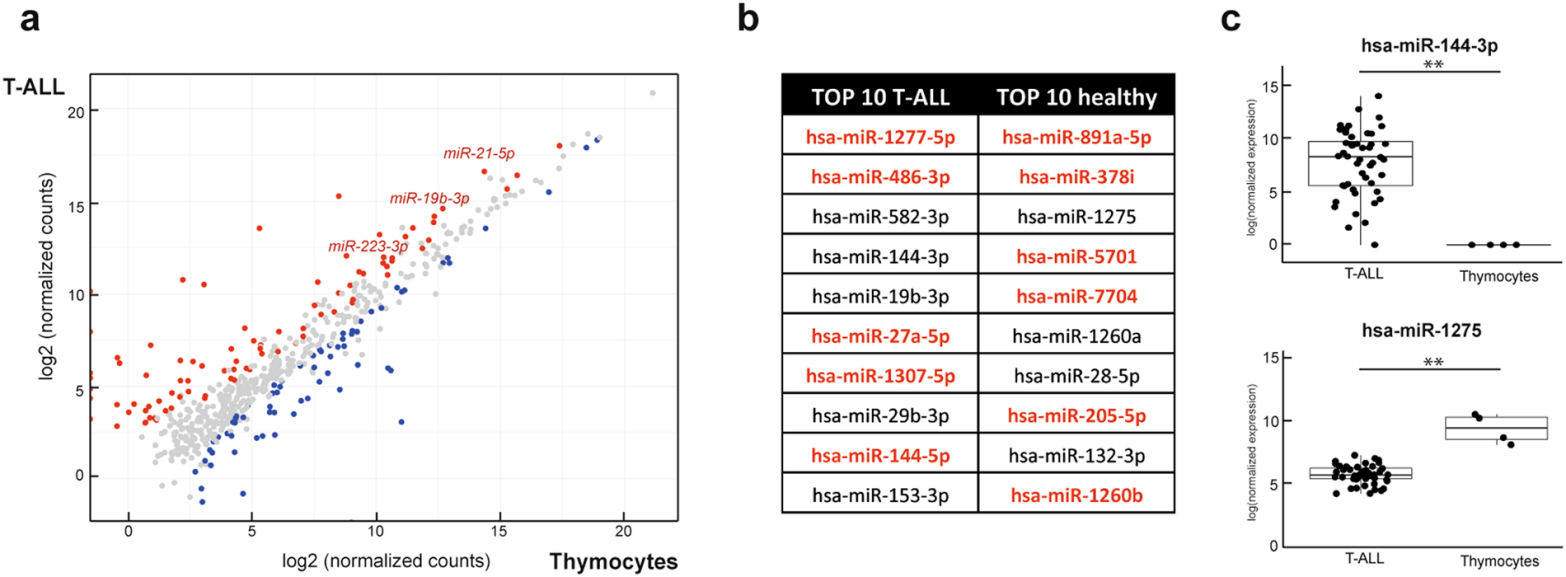
Novel oncogenic miRNAs are detected by small RNA-sequencing. (**a**) Diagonal plot showing the expression of the miRNAs in T-ALL compared to healthy donor thymocytes. Red dots represent miRNAs that are significantly higher expressed in the T-ALL patient samples, blue dots are the miRNAs significantly higher expressed in thymocyte subsets. (**b**) Top 10 most significantly upregulated miRNAs in the T-ALL patients and in the healthy donor samples. MiRNAs denoted in red were not detected by a previously used RT-qPCR platform. (**c**) Dot plots of two representative miRNAs from the table. **Significant difference with an adjusted p-value < 0.001.

Finally, we aimed to identify novel T-ALL miRNAs with potential oncogenic activity in human T-ALL irrespective of the genetic subtypes. Differential expression analysis between 48 T-ALL samples and four normal thymocyte samples (Supplementary Table S5) resulted in the identification of 87 significantly upregulated miRNAs and 69 downregulated miRNAs in human T-ALL (Fig. 5a). Several miRNAs with a known oncogenic role in T-ALL^8, 19^ were recovered from this analysis and are depicted in Fig. 5a. However, and most notably, this analysis also identified different miRNAs, which were not previously detected in the context of normal and malignant T cell development, and which could potentially act as novel oncomiRs or tumor suppressor miRNAs involved in the biology of this disease (Fig. 5b,c).

## Discussion

More than a decade ago, Ferrando *et al*. and Soulier *et al*. described different T-ALL subtypes according to specific transcriptional profiles^1, 2^. Last year, we were able to show that these molecular genetic subtypes of human T-ALL also display unique long non-coding RNA expression signatures^5^. Here, we performed small RNA-sequencing on 48 T-ALL patient samples to finalize the transcriptional characterization of human T-ALL by a comprehensive analysis of miRNA expression in this disease.

Small RNA-sequencing enabled the detection of miRNAs that were not included in a previous RT-qPCR analysis of the same patient population^10^. Although most of the newly detected miRNAs in T-ALL were expressed at very low levels, we also identified a selection of novel T-ALL miRNAs with very high expression levels in T-ALL patient samples, including *hsa-miR-181b-5p*, *hsa-miR-423-3p*, *hsa-miR-486-5p* and *hsa-miR-92b-3p* (Fig. 1b). Interestingly, some of these miRNAs have previously been associated with malignant transformation in different tumor entities. For example, *hsa-miR-181b-5p* is a known oncogene in several cancer types as reviewed by Liu *et al*.^20^. In the context of leukemia, overexpression of *hsa-miR-181b-5p* was shown to enhance proliferation in acute myeloid leukemia by targeting *MLK2*. Furthermore, *hsa-miR-92b-3p* was identified as an oncomiR in glioblastoma by targeting *SMAD*3^21^, which is known to be lost in several cases of pediatric T-ALL^22^, and *PTEN* ^23^, a well-established T-ALL oncosuppressor^24, 25^. In addition, *hsa-miR-92b-3p* is also specifically higher expressed in *TLX1/3* T-ALL compared to the other T-ALL subtypes.

Interestingly, small RNA-sequencing also detects deviations from canonical miRNA sequences. Indeed, our analysis revealed 2139 different isomiR forms, corresponding to 481 miRNAs. Remarkably, for only half of the miRNAs, the canonical form showed the highest expression (Fig. 1e). In addition, most highly expressed isomiRs displayed modifications at their 3′ end, suggesting that these alterations would not affect the miRNA seed sequence and, therefore, have no functional effect on target recognition. Nevertheless, a number of studies have shown that these 3′ modifications might impact target specificity and stability of the miRNA^17, 26^.

Finally, small RNA sequencing of human CD34^+^ and CD4^+^CD8^+^ thymocytes enabled the identification of subtype specific and oncogenic miRNAs in the context of human T-ALL. An interesting example is *hsa-miR-486*. *Hsa-miR-486-5p* is one of the highest expressed newly identified miRNAs in T-ALL (Fig. 1b) and, together with *hsa-miR-486-3p*, it also shows higher expression in T-ALL samples as compared to healthy donor thymocytes. *Hsa-miR-486-5p* is an oncomiR in Down syndrome myeloid leukemias, where it is regulated by GATA1^27^. *Hsa-miR-486-3p* has also been linked to erythroid development downstream of MYB, a known oncogene in T-ALL, and targeting *MAF* ^28^. The differences in miRNA expression could however also be due to the underrepresentation of normal thymocyte differentiation stages in this study. Additional experiments are thus needed to prove the oncogenic nature of the differentially expressed miRNAs.

Altogether, this study provides the first comprehensive overview of miRNA expression in molecular-genetic subtypes of human T-ALL. Integration of these signatures with miRNA expression profiles in normal T-cell subsets provides a unique resource to study novel miRNAs that are implicated in T-ALL disease biology.

## Materials and Methods

### Study design

Small RNA-sequencing was performed on 48 primary T-ALL patient samples, 7 T-ALL cell lines and 2 CD34^+^ and 2 CD4^+^CD8^+^ healthy donor thymocyte subsets, to profile the full T-ALL miRNA transcriptome.

### Primary human T-ALL patient samples and T-ALL cell lines

Blood samples and bone marrow lymphoblast from T-ALL patients were collected after informed consent according to the Declaration of Helsinki from Saint-Louis Hospital, Paris, France. This study was approved by both the Institut Universitair d’Hématologie Institutional Review Board and the Ethical Committee of Ghent University Hospital. Total RNA was isolated using the miRNeasy mini kit (Qiagen). These samples are part of a cohort previously investigated by mRNA^29^ and lncRNA profiling^5, 30^. The T-ALL cell lines LOUCY, DND-41, HPB-ALL, ALL-SIL, PF-382 and JURKAT were purchased from DSMZ. KE-37 was a kind gift from the Cools lab.

### Thymocyte subset selection

Thymus tissue was derived from children undergoing cardiac surgery (UZ Gent) and was obtained and used with informed consent and according to the guidelines of the Medical Ethical Commission of Ghent University Hospital (Ghent, Belgium). Both thymocyte subsets were each purified from different healthy donors in order to obtain two independent replicates for each subset. Immature CD34^+^ thymocytes were purified based on MACS purification using CD34 microbeads (Miltenyi Biotec)^31^ without lineage depletion, while CD4 and CD8 labeling was used to sort the CD4^+^CD8^+^ double positive subset by a FACSAriaIII (BDBiosciences)^32^. The purity of each subset was at least 98%. Total RNA was isolated using the miRNeasy mini kit (Qiagen).

### MicroRNA profiling by small RNA-sequencing

The libraries for small RNA-sequencing were prepared using the TruSeq small RNA library kit from Illumina with 50 ng of total RNA as input for T-ALL samples and 100 ng of total RNA as input for T-ALL cell lines and thymocyte subset samples. According to the manufacturer’s use, 3′ and 5′ RNA adapters were ligated to the RNA followed by reverse transcription and PCR amplification (with bar-coded primers). The PCR products were separated using a Pippin Prep System to recover the 147 nt and 157 nt fractions. Sequencing of the small RNA libraries was performed on a NextSeq500 (Illumina), with an average of 14.4 million reads per sample. After read quality control and adapter trimming, reads were mapped to the reference genome (GRCh38) using Bowtie^33^. Raw data files are submitted into the GEO database^34^ with accession number GSE89978.

### Differential expression analysis

MicroRNA expression data was filtered with a background correction that only retained miRNAs detected by at least 4 reads in at least 60% of samples from one T-ALL subgroup or in at least all samples from one thymocyte subset. Differential expression analysis was performed using the DESeq2 algorithm in R^35^. The expression was normalized using de Variance Stabilizing Transformation from the DESeq2 algorithm.

### Data availability

Reviewers can access the data submitted to the GEO database trough this private link: https://www.ncbi.nlm.nih.gov/geo/query/acc.cgi?token=kzszmioallifrmv&acc=GSE89978.

## Electronic supplementary material

Supplementary info

Supplementary datasets

## References

[1] Soulier J (2005). HOXA genes are included in genetic and biologic networks defining human acute T-cell leukemia (T-ALL). Blood.

[2] Ferrando AA (2002). Gene expression signatures define novel oncogenic pathways in T cell acute lymphoblastic leukemia. Cancer Cell.

[3] Van Vlierberghe P, Pieters R, Beverloo HB, Meijerink JP (2008). Molecular-genetic insights in paediatric T-cell acute lymphoblastic leukaemia. Br J Haematol.

[4] Meijerink JP (2010). Genetic rearrangements in relation to immunophenotype and outcome in T-cell acute lymphoblastic leukaemia. Best Pract Res Clin Haematol.

[5] Wallaert A (2016). Long noncoding RNA signatures define oncogenic subtypes in T-cell acute lymphoblastic leukemia. Leukemia.

[6] Esquela-Kerscher A, Slack FJ (2006). Oncomirs - microRNAs with a role in cancer. Nat Rev Cancer.

[7] Ha M, Kim VN (2014). Regulation of microRNA biogenesis. Nat Rev Mol Cell Biol.

[8] Mavrakis KJ (2011). A cooperative microRNA-tumor suppressor gene network in acute T-cell lymphoblastic leukemia (T-ALL). Nat Genet.

[9] Sanghvi VR (2014). Characterization of a set of tumor suppressor microRNAs in T cell acute lymphoblastic leukemia. Sci Signal.

[10] Mets E (2015). MicroRNA-193b-3p acts as a tumor suppressor by targeting the MYB oncogene in T-cell acute lymphoblastic leukemia. Leukemia.

[11] Neilsen CT, Goodall GJ, Bracken CP (2012). IsomiRs–the overlooked repertoire in the dynamic microRNAome. Trends Genet.

[12] Lee LW (2010). Complexity of the microRNA repertoire revealed by next-generation sequencing. RNA.

[13] Starega-Roslan J, Witkos TM, Galka-Marciniak P, Krzyzosiak WJ (2015). Sequence features of Drosha and Dicer cleavage sites affect the complexity of isomiRs. Int J Mol Sci.

[14] Wyman SK (2011). Post-transcriptional generation of miRNA variants by multiple nucleotidyl transferases contributes to miRNA transcriptome complexity. Genome Res.

[15] Tan GC (2014). 5′ isomiR variation is of functional and evolutionary importance. Nucleic Acids Res.

[16] Derrien T (2012). The GENCODE v7 catalog of human long noncoding RNAs: analysis of their gene structure, evolution, and expression. Genome Res.

[17] Burroughs AM (2010). A comprehensive survey of 3′ animal miRNA modification events and a possible role for 3′ adenylation in modulating miRNA targeting effectiveness. Genome Res.

[18] Van Peer, G. *et al*. miRBase Tracker: keeping track of microRNA annotation changes. *Database (Oxford)* **2014**, doi:10.1093/database/bau080 (2014). 10.1093/database/bau080 PMC4142392 25157074

[19] Mavrakis KJ (2010). Genome-wide RNA-mediated interference screen identifies miR-19 targets in Notch-induced T-cell acute lymphoblastic leukaemia. Nat Cell Biol.

[20] Liu J, Shi W, Wu C, Ju J, Jiang J (2014). miR-181b as a key regulator of the oncogenic process and its clinical implications in cancer (Review). Biomed Rep.

[21] Wu ZB (2013). The miR-92b functions as a potential oncogene by targeting on Smad3 in glioblastomas. Brain Res.

[22] Wolfraim LA (2004). Loss of Smad3 in acute T-cell lymphoblastic leukemia. N Engl J Med.

[23] Song H (2016). miR-92b regulates glioma cells proliferation, migration, invasion, and apoptosis via PTEN/Akt signaling pathway. J Physiol Biochem.

[24] Zuurbier L (2012). The significance of PTEN and AKT aberrations in pediatric T-cell acute lymphoblastic leukemia. Haematologica.

[25] Silva A (2008). PTEN posttranslational inactivation and hyperactivation of the PI3K/Akt pathway sustain primary T cell leukemia viability. J Clin Invest.

[26] Lu S, Sun YH, Chiang VL (2009). Adenylation of plant miRNAs. Nucleic Acids Res.

[27] Shaham L (2015). MicroRNA-486-5p is an erythroid oncomiR of the myeloid leukemias of Down syndrome. Blood.

[28] Bianchi E (2015). MYB controls erythroid versus megakaryocyte lineage fate decision through the miR-486-3p-mediated downregulation of MAF. Cell Death Differ.

[29] Clappier E (2011). Clonal selection in xenografted human T cell acute lymphoblastic leukemia recapitulates gain of malignancy at relapse. J Exp Med.

[30] Durinck K (2014). The Notch driven long non-coding RNA repertoire in T-cell acute lymphoblastic leukemia. Haematologica.

[31] Van de Walle I (2013). Specific Notch receptor-ligand interactions control human TCR-alphabeta/gammadelta development by inducing differential Notch signal strength. J Exp Med.

[32] Taghon T, Waegemans E, Van de Walle I (2012). Notch signaling during human T cell development. Curr Top Microbiol Immunol.

[33] Langmead B, Trapnell C, Pop M, Salzberg SL (2009). Ultrafast and memory-efficient alignment of short DNA sequences to the human genome. Genome Biol.

[34] Edgar R, Domrachev M, Lash AE (2002). Gene Expression Omnibus: NCBI gene expression and hybridization array data repository. Nucleic Acids Res.

[35] Love MI, Huber W, Anders S (2014). Moderated estimation of fold change and dispersion for RNA-seq data with DESeq. 2. Genome Biol.

