# Supplementary material for: Comprehensive miRNA expression profiling in human T-cell acute lymphoblastic leukemia by small RNA-sequencing
Source: Sci Rep. 2017 Aug 11;7:7901. doi: 10.1038/s41598-017-08148-x (PMC5554241; doi:10.1038/s41598-017-08148-x)
Supplement: Supplementary file 1 — Supplementary info [file 41598_2017_8148_MOESM1_ESM.pdf]

Comprehensive miRNA expression profiling in human T-cell acute lymphoblastic leukemia by small RNA-sequencing

Annelynn Wallaert, Wouter Van Loocke, Lucie Hernandez, Tom Taghon, Frank Speleman and Pieter Van Vlierberghe

Supplementary Figure S1

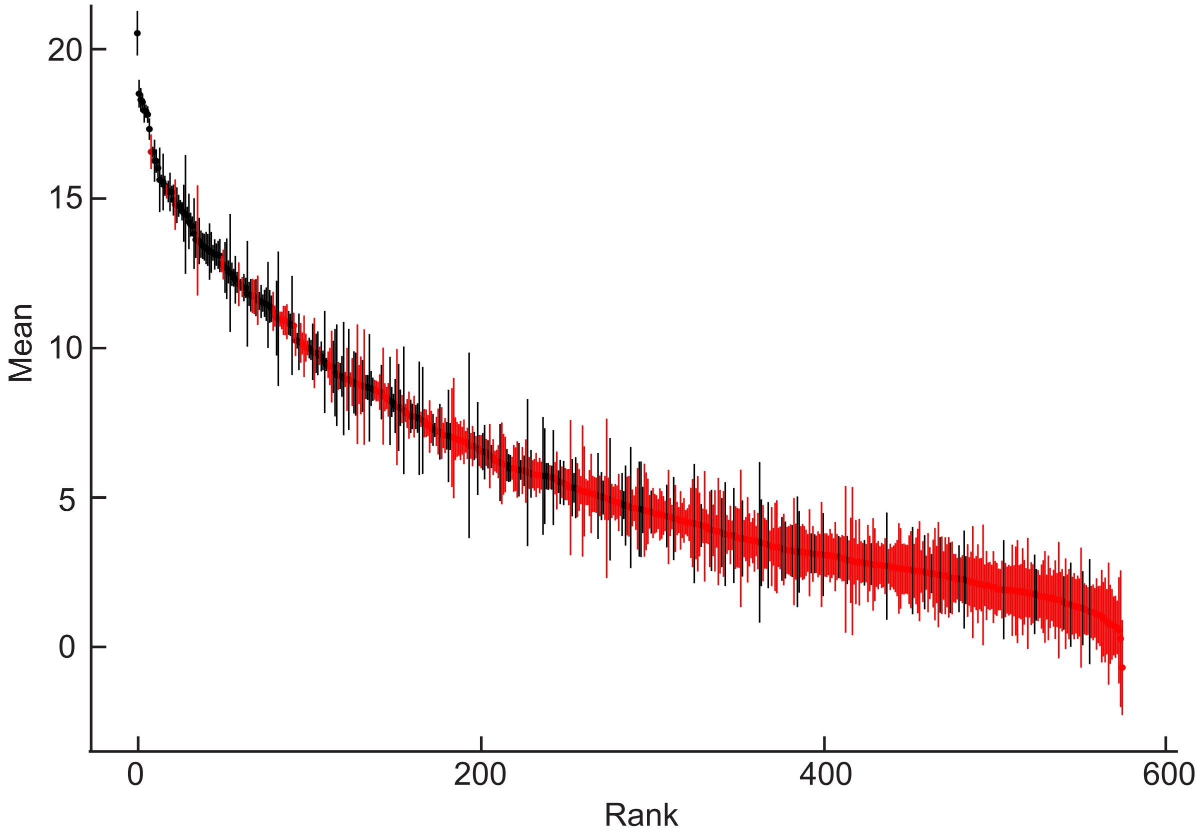

Supplementary Figure S1. Dot plot with standard deviation representing the mean normalized expression levels of all 574 miRNAs detected by small RNA-seq. The miRNAs are ranked from highest to lowest mean expression. Red dots/bars represent miRNAs that were not detected by an RT-qPCR platform from previous studies.

Supplementary Figure S2

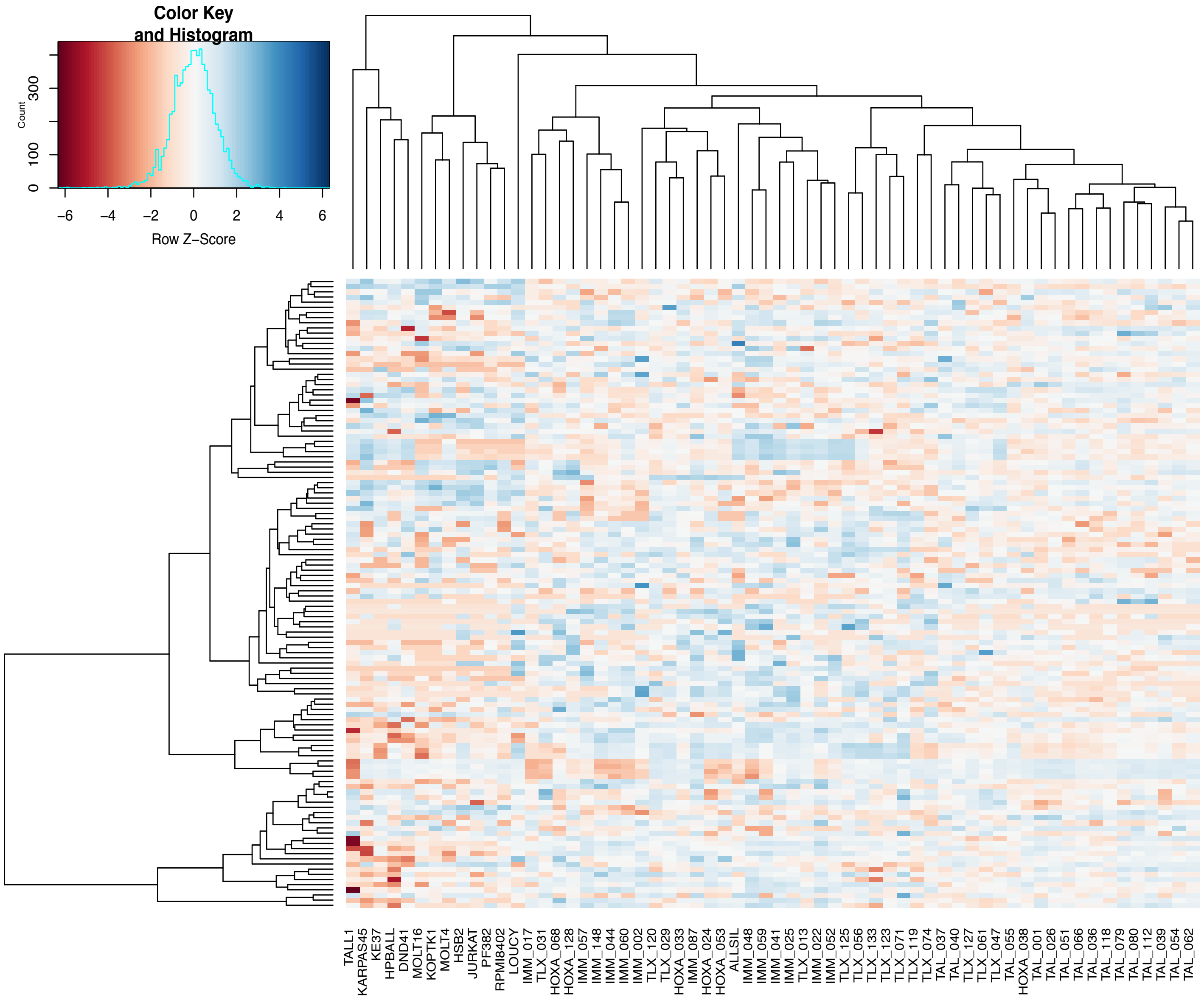

Supplementary Figure S2. Clustering of T-ALL patient samples and T-ALL cell lines using the expression of the T-ALL subtype specific miRNAs.

Supplementary Figure S3

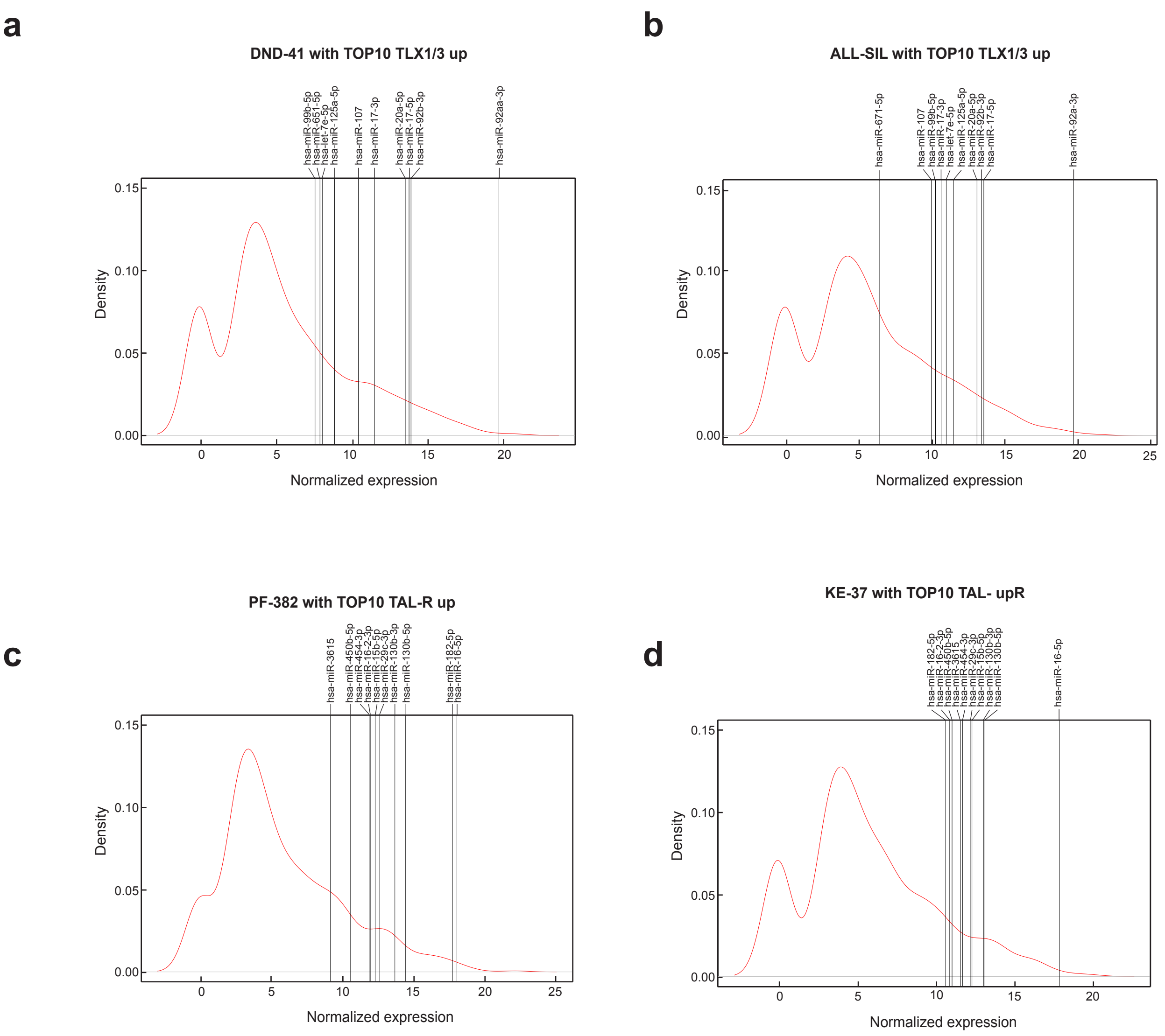

Supplementary Figure S3. Subtype specific miRNAs are also represented in the T-ALL cell lines. Density plots representing the distribution of the miRNA expression in the T-ALL cell lines DND-41, ALL-SIL, PF-382 and KE-37. Vertical bars show the expression level of the top 10 miRNAs selected for the subgroup these cell lines represent. DND-41 (A) and ALL-SIL (B) for the TLX subgroup and PF-382 (C) and KE-37 (D) for the TAL/LMO subgroup.

Supplementary Figure S4

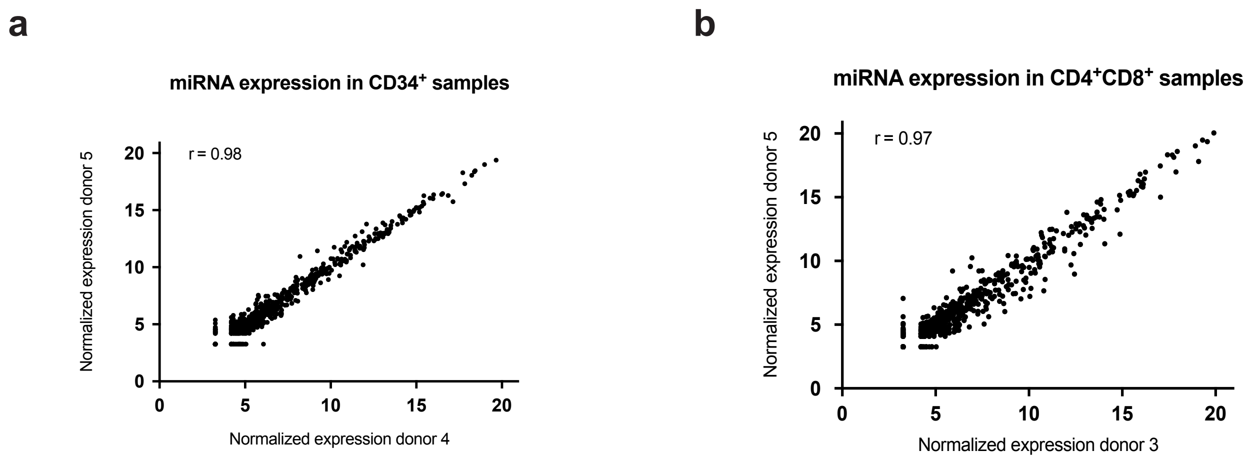

Supplementary Figure S4. miRNA expression is highly correlated in 2 different donors. Scatterplots representing the log2 normalized expression of the miRNAs profiled by small RNA-sequencing for 2 different donors of CD34+ thymocytes (A) and $CD4^{+}CD8^{+}$ thymocytes (B).
